# Evaluation of safety boots and their relationships with the foot structure of female and male industrial workers

**DOI:** 10.1186/s12891-025-08983-4

**Published:** 2025-10-01

**Authors:** Ewa Puszczalowska-Lizis, Sabina Lizis, Wioletta Mikulakova, Dorota Kubicz, Lucia Demjanovic Kendrova, Jaroslaw Omorczyk

**Affiliations:** 1 Institute of Physiotherapy, Faculty of Health Sciences and Psychology, Collegium Medicum, University of Rzeszow, Rejtana 16 C Av., 35-959 Rzeszow, Poland; 2 Department of Physiotherapy, Faculty of Health Care, University of Presov, Partizanska 1 St, Presov, 080 01 Slovakia; 3 Physiotherapy Unit, Non Public Physiotherapy Practice, Strazacka 12St., 35-312, Rzeszow, Poland; 4 Institute of Sport Sciences, Department of Gymnastics and Dance, University of Physical Culture in Krakow, John Paul II 78 Av., 31-571 Krakow, Poland

**Keywords:** Foot dimension, Foot deformities, Shoe fit, Workplace, Production floors

## Abstract

**Background:**

Working conditions and the variety of activities performed in different occupations require footwear that meets the safety needs of the worker. More and more females are entering the industrial environment, which until recently was dominated by males, so there are indications of problems in adapting safety footwear to female feet. The aim of this research was to evaluate foot characteristics as well as perceived comfort, functionality and attractiveness of safety boots and their mutual relationships among production workers.

**Methods:**

The study included 80 industrial workers aged 50–60 years (35 females; 45 males). The foot was evaluated using the CQ-ST Podoscope. Perception of comfort, functionality and attractiveness of GALMAG safety boots were evaluated. The research results were analyzed using Student’s t-test for independent variables, Mann-Whitney U-test and Spearman’s rank correlation.

**Results:**

Statistically significant gender differences were found in terms of individualization (*p* = 0.044) and attractiveness of shoes (*p* = 0.041), as well as relationships of right (*p* = 0.010) and left (*p* = 0.030) foot width with shoe length perception, and Clarke’s angle of the right (*p* = 0. 004) and left (*p* = 0.020) foot with heel cushioning, Clarke’s angle of the right (*p* = 0.020) and left (*p* = 0.003) foot with shoe weight ratings, and Clarke’s angle of the right (*p* = 0.007) and left (*p* = 0.029) foot with shoe breathability. Clarke’s angle of the right (*p* = 0.020) and left (*p* = 0.049) foot also correlated with ratings of shoe style, and β angle of the right foot with shoe weight (*p* = 0.042) and breathability (*p* = 0.043).

**Conclusions:**

It is necessary to develop recommendations for designers and manufacturers of safety boots to move away from the production of unisex footwear and the use of female’s lasts in the production of female footwear. Different widths for the same length should be considered when designing safety boots. People with high arches need better cushioning and therefore prefer insoles made of soft, flexible materials. More comfortable footwear is also more functional and aesthetically pleasing. Therefore, considering these aspects in the design and manufacture of footwear can result in the proper functioning of the worker in the workplace.

**Trial registration:**

Not required, as the study design envisaged no health interventions whatsoever.

## Background

Work in the manufacturing, construction, civil engineering and mining sectors has a high risk of exposure to hazardous and harmful agents. Therefore, employers are required to provide workers with personal protective equipment, including appropriately designed footwear called safety footwear. The primary function of these shoes is to protect the distal parts of the lower extremities, including the feet, from the effects of external factors such as contamination, soaking, burning, cutting, impact and crushing. The safety degree is determined by the design and materials used to make the footwear. The level of protection can be increased by elements such as steel toecaps, reflective elements, oil-resistant soles or shock absorbers. The requirements and criteria that protective footwear must meet are defined in safety standards [1–4]. The standard currently in force in Europe is EN ISO 20345. Based on the level of risk, safety footwear is divided into: safety shoes (with metal or composite toecaps capable of withstanding impacts up to 200 J), protective shoes (with toecaps limited to 100 J) and occupational shoes, also known as work shoes (used to protect against injury when working in areas where toe protection with toecaps is not required) [4, 5]. Dobson et al. [6] and Kobayashia et al. [7] pointed out that shoes with steel toecaps in the front of the upper, while providing toe protection, may restrict toe movement during locomotion. They are also much heavier than regular shoes.

Working conditions and the variety of activities performed in different occupations require the use of different footwear designs to meet the safety needs of workers. It should be noted that in addition to protection against safety hazards, functionality and comfort are both important, which can be related to the fit of the footwear to the shape of the foot. This is particularly important for people with foot deformities and for females. Krauss et al. [8–10] highlighted sex-related differences in foot structure. In fact, Cauley [11] emphasized that the feet of females are more delicate, both in terms of the bony elements’ size and the strength of the active and passive stabilizers. Consequently, contact of the foot with footwear may cause reactions to differ among women and men [12, 13]. Janson et al. [14] noted that an increasing number of females are entering industrial environments that, until recently, have been dominated by males. There is reason to believe that there may be problems with the fit of safety footwear to female’s feet, thus it may be considered uncomfortable. The authors also pointed out the tendency of female to focus on aesthetic qualities. Meanwhile, Anderson et al. [15] showed that most people working in demanding environments find their work shoes unattractive. The appearance of a shoe conveys perceptions about it, including those related to its function, performance and ergonomic quality. Additionally, Krauss et al. [16] pointed out that knowledge of sex-related differences in foot measures is important to assist proper shoe fit in both men and women. Important information can be provided by research on the connections between the assess-ment of comfort, functionality and attractiveness of footwear. Knowledge of these correlations will allow us to design footwear meeting the expectations of users in terms of aesthetics while, at the same time, providing comfort and being practical in use, which will certainly translate into improved foot health and user well-being. There is a lack of studies in the literature that address these issues in relation to the use of contoured models of safety footwear. This was the immediate reason for the study, whose aim was to evaluate foot characteristics as well as perceived comfort, functionality and attractiveness of safety boots and their mutual relationships among production workers.

Research questions:

1. Do foot characteristics as well as opinions on the comfort, functionality and style of evaluated safety boots differentiate female and male industrial workers?
2. What are the relationships between foot characteristics and ratings of safety boot comfort, functionality and style?
3. What are the relationships between comfort perceptions and footwear functionality as well as attractiveness of the evaluated safety boots?

## Methods

### Participants

The study, conducted in May 2023, involved 80 occupationally active production workers aged 50–60 years, including 35 females (44% of the group) and 45 males (56% of the group) employed in one of the randomly selected industrial plants in the Podkarpackie Voivodeship, Poland. The sample size representative for the site was estimated considering a 95% confidence level, and a 5% level of fraction estimation admissible error. The mean age of the females was 55.09 ± 3.12 years and of the males 52.76 ± 3.34 years.

The following study inclusion criteria were used: work in the production hall of an industrial plant; age 50–60 years; wearing S1 safety boots at work for 7 days prior to the study, for at least 7 h a day; written informed consent to participate in the study. Excluded from recruitment were those with a recent history of musculoskeletal trauma and surgery, and those who refused or declined to participate in the study.

The data in Table 1 allow to indicate that the females had lower body mass and height values, whereas no statistically significant sex-related differences were found in terms of BMI.

**Table 1:** Sex-related comparison of bodily characteristics

Group	x̄± SD	Max-Min	Q_25_	Me	Q_75_	t/Z	p
Body mass [kg]							
Female	73.49±12.73	98.00-50.00	63.00	74.00	80.00	t=-4.52	<0.001*
Male	87.53±14.51	130.00-64.00	75.00	86.00	95.00		
Body height [cm]							
Female	164.34±6.12	178.00-152.00	160.00	164.00	168.00	Z=-6.51	<0.001*
Male	176.96±6.40	194.00-163.00	173.00	176.00	180.00		
BMI							
Female	27.29±5.08	37.50-20.03	22.59	26.93	31.60	Z=-0.70	0.485
Male	27.93±4.31	40.83-22.15	24.34	27.04	30.11		

*Abbreviations*: $\bar{x}$ Arithmetical mean value, *SD *Standard deviation, *Max *Maximum value, *Min* Minimum value, *Q*_25_ Lower quartile, *Me* median, *Q*_75_ Upper quartile,

t– value of the Student's t test for independent variables

Z– value of the Mann Whitney U test statistic

p– probability value

* p<0.05

### Design

Foot examination was performed by podoscopic method. The research tool was CQ-ST podoscope manufactured by Electronic System, Ltd., EU. During the measurement, the subject was in a free standing position, with the body weight evenly distributed on each foot. The following indices were analyzed:

1. Foot length [cm] measured between distal points of the forefoot and hindfoot (line L);
2. Foot width [cm] measured between metatarsale tibiale (mtt) and the metatarsale fibulare (mtf) points (line W);
3. Clarke’s angle [°]– medial longitudinal foot arch– measured between the tangent to the medial foot edge and the line that connect mtt point with the largest recess of the foot (Cl angle);
4. Heel angle [°]– transverse foot arch– measured between the tangents to the medial and lateral edge of the foot which cross over the heel (γ angle);
5. Hallux valgus angle [°] measured between the tangents to the medial foot edge, and to the pad of the Ist toe, marked from the mtt point (α angle);
6. Angle of the varus deformity of the Vth toe [°] measured between the tangents to the lateral foot edge, and the pad of the Vth toe (β angle), marked from the mtf point [13, 17, 18].

**Fig. 1:**
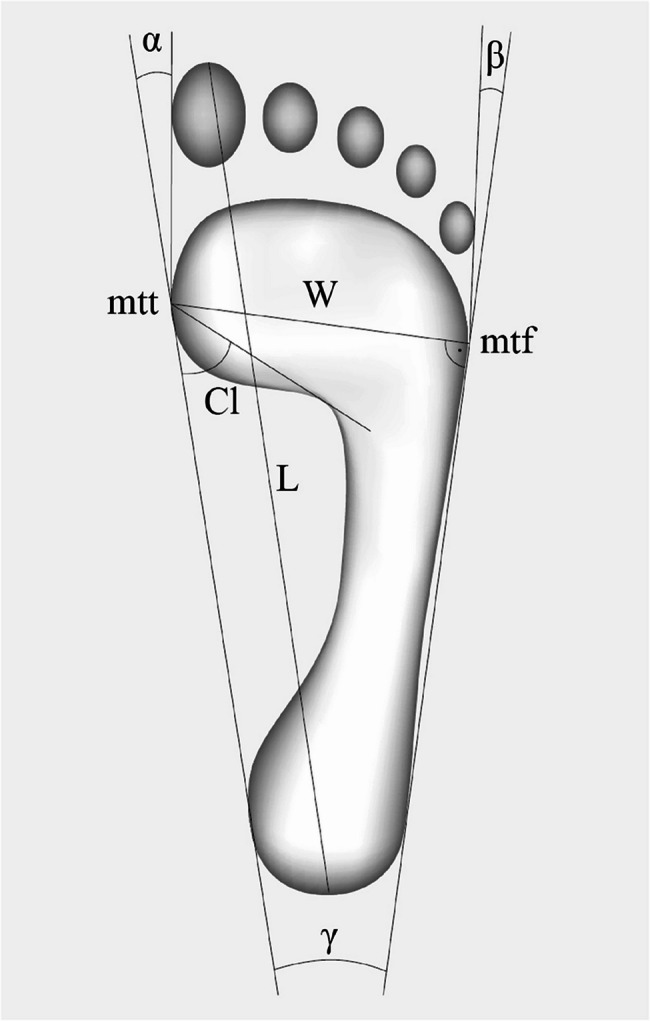
The manner of marking the foot structure indices

The decision to choose such measures was determined by the choice of the research tool, which was a podoscope. Moreover, we additionally included the selection of indicators that can be found in extensive literature on the subject. This index rate was amongst the most commonly used to foot evaluation. The manner of marking the above indices is presented in Fig. 1.

**Fig. 2:**
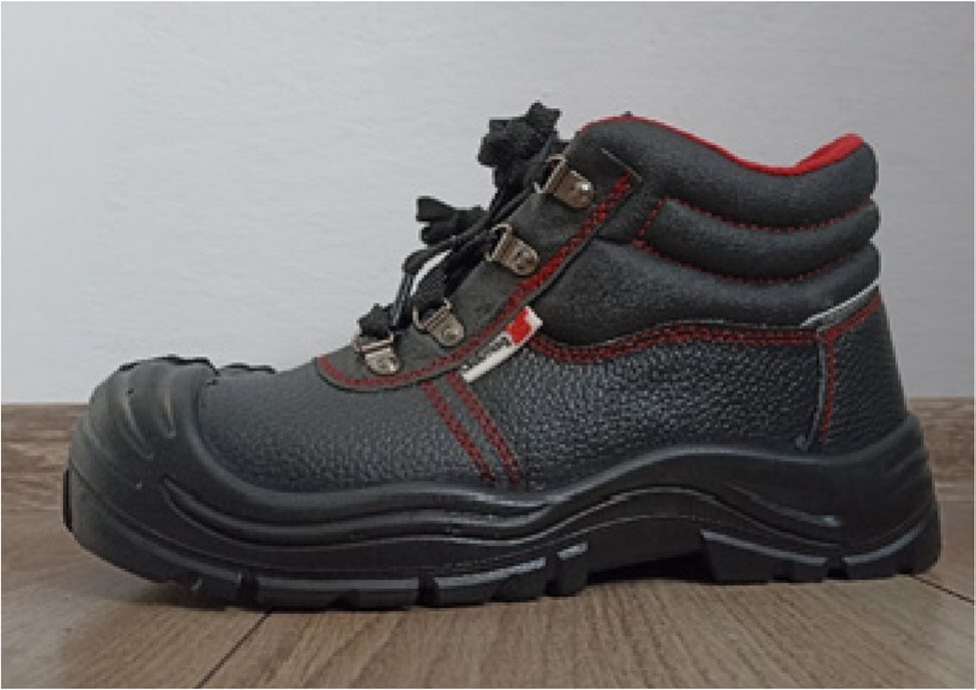
GALMAG safety boots evaluated in the research

The perception of comfort, functionality and attractiveness of footwear for people working in production areas was assessed. There were GALMAG safety boots (Fig. 2), manufactured by Galmag, Poland, EU (manufacturer code: 471R42) with steel toecap for protection against mechanical injuries. These are “unisex” shoes. The upper of the boots is made of 1.4-1.6 mm thick calf leather with synthetic inserts. The lining is made of mesh material with hygroscopic properties. The footwear has a closed heel, energy absorption in the heel and antistatic properties. They have a soft EVA (ethylene vinyl acetate) polyamide insole. The sole has anti-slip properties and is also resistant to acidic and oily substances, petrol and other organic solvents. The shoes comply with the safety standards for protective footwear (EN ISO 20345:2011) and are classified as S1, which is characterized by the energy absorption capacity of the metal toe cap, which can withstand an impact of 200 J and a crushing force of up to 15 kN.

The subjects wore these shoes at work for at least seven hours a day for seven days prior to the study. Although employees had worn this type of footwear before being recruited for the study, new shoes were provided by the employer for all participants free-of-charge and specifically for the purposes of the research. The shoes were supplied in a size that corresponded to the length of the feet. Before the test began, the researchers also checked the fit of the shoes on the test feet while the participants stood in an upright, equally loaded position. If the toes could move freely in the forefoot and the heel rested firmly on the heel counter, the shoe was considered suitable.

The evaluation of the comfort of the safety boots was carried out according to the criteria proposed by Mündermann et al. [19], which included the following categories: Shoe length; Shoe forefoot width (width of the shoe in the forefoot area); Shoe heel width (width of the shoe in the heel area); Heel height (height at which the rear foot is raised in relation to the forefoot); Heel cushioning (softness/hardness of the midsole in the heel area); Forefoot cushioning (softness/hardness of the midsole in the forefoot area); Arch height (medial arch height of the insole); Mediolateral control (position of the foot controlled by the shoe); Overall comfort (overall impression of the shoe). The reliability index of this research tool totals ICC = 0.799[19]. The subject rated the information on a 10-point scale, with the left end being"not comfortable at all" (0 points) and the right end being"most comfortable" (10 points). In addition, another structural element of the shoe that influences comfort perception was assessed, such as"material properties of the shoe", which depends on the quality/amount of fabrics used in the construction and the type of stitching. 

The evaluation of the functionality of safety boots was carried out using the criteria proposed by Anderson et al. [20], which included the following categories: Grip (adhesion of the footwear to the ground, resistance of the footwear to sliding on the ground); Durability (resistance of the footwear to damage); Safety (protection of the feet from injuries caused by heavy or sharp objects, liquid spills, etc.); Weight (weight of the footwear); Breathability (ability to drain evaporated sweat to the outside of the footwear); Ease of donning and doffing (solutions for quick donning and doffing); Individualiztion (fitting of the footwear to the foot). The subject rated the information on a 10-point scale, with the left end of the scale being "not functional at all" (0 points) and the right end of the scale being "most functional" (10 points).

Evaluation of safety shoe style based on design, appearance, attractiveness, and presence of the shoe on the leg [20] on a 10-point scale with the left end labeled"unattractive" (0 points) and the right end labeled "most attractive" (10 points).

Using the same 10-point scale for all the mentioned research tools allowed unification of data analysis. Perceptions of shoe comfort, functionality, and style were rated by the participants in the presence of the investigator after being fully informed of all shoe issues and how to score responses on a visual analog scale. Further explanation was provided as needed.

A study protocol was endorsed by the Bioethics Review Committee at University of Rzeszow (Appr. Ref. No. 3/12/2015). The study was completely anonymous and conducted in accordance with the tenets of the Declaration of Helsinki. Each subject gave written informed consent to participate after receiving a detailed explanation of the research, including information about the purpose of the study, how the data would be collected, the participant's right to withdraw at any time, and the anonymity and confidentiality of the data, with the following content: „I…… voluntarily agree to participate in the research study. I have read and understood the study information. The purpose and nature of the study was explained to me. I have been able to ask questions about the study and my questions have been answered to my satisfaction. I consent voluntarily that I am a participant in this study and I understand that I can refuse to participation and I can withdraw from the study at any time, without giving a reason. I understand that all information I provide for this study will be treated confidentially. I understand that in all of reports on the results of this research my identity will remain anonymous”.

### Analysis

The Shapiro-Wilk test was used to assess whether a data set was normally distributed. Sex-related comparison of bodily and foot characteristics as well as variables determining perceptions of comfort, functionality and attractiveness of safety boots were calculated using the Student's t-test for independent variables or, alternatively, the non-parametric Mann-Whitney U test. Spearman's rank correlation was used to analyze relationships of foot characteristics with ratings of safety boots comfort, functionality and style, as well as relationships of comfort perceptions with footwear functionality and attractiveness ratings. The significance level was set at 5%. Statistics were performed using Statistica v.13.3 PL application (StatSoft Inc., Tulsa, OK, USA; StatSoft, Krakow, Poland).

## Results

The data in Table 2 show statistically significant differences in the foot length and width of the females and males studied. Females feet were characterized by lower values of these features. No statistically significant intersex differences were found for the other indices. 

**Table 2:** Sex-related comparison of foot characteristics

Variable		Female			Male			Z	p
		x̄±SD	Max-Min	Me	x̄± SD	Max-Min	Me		
Foot length	rf	22.68±1.00	25.00-20.50	22.50	25.03±1.09	28.00-22.50	25.00	-6.93	<0.001*
	lf	22.74±1.00	25.00-20.60	22.70	25.04±1.09	28.00-22.50	25.00	-6.87	<0.001*
Foot width	rf	8.88±0.44	9.70-7.80	8.90	9.49±0.55	11.00-8.30	9.50	-4.78	<0.001*
	lf	8.89±0.51	10.00-7.80	9.00	9.45±0.55	10.70-8.30	9.50	-4.03	<0.001*
Cl angle	rf	37.43±10.29	52.00-10.00	40.00	37.93±9.32	52.00-9.00	40.00	0.05	0.957
	lf	37.43±10.82	59.00-6.00	40.00	41.13±8.62	53.00-9.00	42.00	-1.70	0.089
γ angle	rf	17.63±1.90	22.00-15.00	17.00	17.42±1.71	23.00-15.00	18.00	0.40	0.688
	lf	17.60±1.72	20.00-15.00	18.00	17.38±1.90	22.00-14.00	17.00	0.77	0.442
α angle	rf	7.91±5.77	25.00-0.00	7.00	7.18±4.84	19.00-0.00	6.00	0.37	0.712
	lf	8.74±6.55	26.00-0.00	7.00	6.29±4.88	16.00-0.00	5.00	1.59	0.113
β angle	rf	20.51±4.69	29.00-12.00	20.00	19.71±5.12	32.00-9.00	20.00	0.80	0.422
	lf	18.46±5.05	27.00-8.00	18.00	19.31±4.83	30.00-6.00	20.00	-0.67	0.502

*Abbreviations*: *rf *right foot, *lf *left foot, $\bar{x}$ arithmetical mean value, *SD* standard deviation, *Max* maximum value, *Min *minimum value, *Me* median

Z– value of the Mann Whitney U test statistic

p– probability value

*p<0.05

The data in Table 3 show that there were no statistically significant gender differences in the values of the variables that determine the perceived comfort of the tested footwear. A comparison of the variables for the functionality of safety footwear showed statistically significant gender differences in terms of individualization (p=0.044). Females were characterized by lower values of the mentioned characteristics. The values of the variable determining the attractiveness of the footwear were also lower in the females group than in the males group (p=0.041).

**Table 3:** Sex-related comparison of variables determining perceptions of comfort, functionality and attractiveness of safety boots

Variable	Female			Male			Z	p
	x̄±SD	Max-Min	Me	x̄±SD	Max-Min	Me		
Perception of safety boots comfort [points]								
Shoe length	7.14±1.99	10.00-3.00	7.00	6.27±2.30	10.00-0.00	7.00	1.60	0.109
Shoe forefoot width	6.17±2.28	10.00-3.00	5.00	5.67±2.40	10.00-0.00	5.00	0.66	0.510
Shoe heel width	6.54±2.27	10.00-3.00	7.00	6.09±1.65	9.00-3.00	6.00	0.88	0.377
Heel height	6.80±2.36	10.00-0.00	7.00	6.40±1.71	10.00-1.00	7.00	1.18	0.240
Heel cushioning	5.37±2.30	10.00-2.00	5.00	4.89±2.18	10.00-1.00	5.00	0.90	0.369
Forefoot cushioning	5.09±2.05	10.00-1.00	5.00	4.93±2.24	10.00-0.00	5.00	0.26	0.799
Arch height	6.17±1.89	10.00-3.00	6.00	6.18±1.76	10.00-2.00	6.00	-0.03	0.972
Mediolateral control	5.97±2.01	10.00-3.00	6.00	5.78±2.08	10.00-2.00	6.00	0.30	0.765
Overall comfort	5.20±2.30	10.00-1.00	5.00	5.18±2.36	10.00-0.00	5.00	-0.14	0.891
Material properties	4.80±2.30	10.00-1.00	4.00	4.87±2.45	10.00-0.00	5.00	-0.46	0.645
Functionality of safety boots [points]								
Grip	6.46±1.88	10.00-3.00	7.00	6.60±2.02	10.00-2.00	7.00	-0.29	0.771
Durability	5.91±1.93	10.00-2.00	5.00	6.04±2.20	10.00-0.00	6.00	-0.68	0.497
Safety	7.03±1.85	10.00-4.00	7.00	7.02±1.57	10.00-4.00	7.00	0.06	0.949
Weight	5.83±2.94	10.00-1.00	6.00	5.27±2.73	10.00-0.00	5.00	0.97	0.333
Breathability	4.51±2.41	10.00-1.00	4.00	4.22±2.31	9.00-0.00	4.00	0.25	0.802
Ease of donning and doffing	6.77±2.21	10.00-3.00	7.00	6.64±1.71	10.00-3.00	7.00	0.17	0.863
Individualiztion	4.43±2.15	10.00-1.00	4.00	5.22±2.29	10.00-0.00	5.00	-2.02	0.044*
Attractiveness of safety boots [points]								
Style	4.09±1.92	10.00-1.00	4.00	4.91±2.15	9.00-0.00	5.00	-2.04	0.041*

*Abbreviations*: $\bar{x}$ arithmetical mean value, *SD* standard deviation, *Max *maximum value, *Min *minimum value, *Me *median

Z– value of the Mann Whitney U test statistic

p– probability value

*p<0.05

The data in Table 4 show statistically significant relationships of right and left foot width with shoe length perception and Clarke's angle of the right and left foot with heel cushioning. The negative direction of these relationships indicates that the higher the values of foot width, the lower the values of comfort points assessing shoe length perception, and also the higher the values of Clarke's angle, the lower the values of comfort points assessing heel cushioning.

**Table 4:** Relationships of foot characteristics with ratings of safety boots comfort

Variable		Shoe length	Shoe forefoot width	Shoe heel width	Heel height	Heel cushioning	Forefoot cushioning	Arch height	Mediolateral control	Overall comfort	Material properties
		R p									
Foot length	rf	-0.18 0.105	-0.05 0.682	-0.10 0.396	-0.12 0.298	-0.15 0.179	0.05 0.650	0.05 0.680	0.02 0.881	0.07 0.529	0.06 0.575
	lf	-0.18 0.114	-0.04 0.701	-0.12 0.300	-0.12 0.289	-0.16 0.161	0.05 0.636	0.05 0.684	0.02 0.862	0.07 0.528	0.05 0.679
Foot width	rf	-0.29 0.010*	-0.15 0.198	-0.18 0.105	-0.18 0.110	-0.21 0.057	-0.06 0.596	-0.05 0.688	-0.12 0.309	-0.07 0.555	-0.10 0.377
	lf	-0.24 0.030*	-0.11 0.319	-0.19 0.099	-0.13 0.261	-0.19 0.089	-0.04 0.696	-0.05 0.668	-0.13 0.266	-0.09 0.439	-0.17 0.134
Cl angle	rf	-0.04 0.725	-0.00 0.980	-0.14 0.212	-0.05 0.649	-0.32 0.004*	-0.18 0.109	-0.14 0.209	-0.09 0.408	-0.17 0.128	-0.15 0.184
	lf	-0.03 0.816	0.02 0.840	-0.15 0.193	-0.01 0.964	-0.26 0.020*	-0.05 0.671	-0.03 0.804	-0.05 0.636	-0.08 0.501	-0.14 0.222
γ angle	rf	0.03 0.811	-0.03 0.761	0.04 0.747	0.03 0.806	0.04 0.734	-0.09 0.408	0.07 0.548	0.06 0.582	-0.02 0.840	-0.09 0.441
	lf	-0.10 0.392	-0.08 0.480	-0.07 0.518	-0.05 0.670	-0.12 0.291	-0.16 0.163	-0.02 0.860	-0.11 0.350	-0.15 0.174	-0.22 0.054
α angle	rf	-0.05 0.681	0.03 0.760	-0.10 0.377	-0.03 0.810	0.18 0.106	0.15 0.185	0.13 0.235	0.08 0.474	0.01 0.926	0.03 0.761
	lf	-0.15 0.190	0.03 0.796	0.02 0.834	0.10 0.371	0.03 0.776	0.01 0.930	0.08 0.485	-0.03 0.812	-0.12 0.309	-0.04 0.733
β angle	rf	0.09 0.422	-0.03 0.792	0.01 0.961	0.03 0.814	-0.13 0.248	-0.16 0.156	-0.06 0.590	-0.05 0.632	-0.09 0.429	-0.19 0.098
	lf	-0.13 0.250	-0.18 0.120	-0.15 0.175	0.04 0.756	-0.16 0.148	-0.17 0.133	-0.08 0.464	-0.07 0.553	-0.16 0.148	-0.07 0.513

*Abbreviations*: *rf *right foot, *lf *left foot, *R *Spearman’s rank correlation coefficient, *p *probability value

*p<0.05

The data in Table 5 show statistically significant relationships of Clarke's angle of the right and left foot with ratings of shoe weight, and Clarke's angle of the right and left foot with ratings of shoe breathability. Statistically significant relationships were also found between Clarke's angle of the right and left foot with ratings of shoe attractiveness (style). The negative direction of the aforementioned relationships indicates that the higher the values of Clarke's angle, the lower the ratings of weight, breathability and attractiveness of footwear.

There was a statistically significant negative relationship between the width of the left foot and the values of the comfort points assessing the ease of donning and doffing. The wider the left foot, the greater the difficulty of donning and doffing.

There was a statistically significant negative relationship of γ angle with shoe weight ratings, meaning that higher γ angle values were associated with lower shoe weight ratings.

There were statistically significant negative relationships of right foot β angle with shoe weight and breathability. The higher the β angle, the lower the shoe weight and breathability ratings.

**Table 5:** Relationships of foot characteristics with ratings of safety boots functionality and style

Variable		Grip	Durability	Safety	Weight	Breathability	Ease of donning and doffing	Individualiztion	Style
		R p							
Foot length	rf	-0.00 0.979	0.01 0.896	-0.04 0.697	-0.10 0.389	-0.00 0.986	-0.04 0.709	0.21 0.058	0.19 0.099
	lf	-0.01 0.951	0.03 0.815	-0.04 0.705	-0.11 0.353	-0.01 0.932	-0.05 0.636	0.21 0.067	0.18 0.118
Foot width	rf	-0.08 0.508	-0.09 0.434	-0.14 0.228	-0.14 0.210	-0.15 0.197	-0.18 0.119	0.00 0.987	-0.00 0.974
	lf	-0.08 0.504	-0.07 0.535	-0.15 0.197	-0.15 0.177	-0.12 0.298	-0.22 0.046*	-0.01 0.899	0.00 0.973
Cl angle	rf	-0.18 0.101	-0.19 0.091	-0.15 0.177	-0.26 0.020*	-0.30 0.007*	0.01 0.935	-0,11 0.325	-0.26 0.020*
	lf	-0.05 0.679	-0.05 0.685	-0.04 0.711	-0.32 0.003*	-0.24 0.029*	-0.04 0.708	-0.06 0.573	-0.22 0.049*
γ angle	rf	0.07 0.531	0.10 0.364	0.11 0.333	-0.09 0.412	0.01 0.941	-0.02 0.888	-0.04 0.693	0.01 0.924
	lf	-0.02 0.860	-0.08 0.487	-0.07 0.554	-0.22 0.049*	-0.17 0.137	-0.22 0.053	-0.20 0.082	-0.19 0.088
α angle	rf	0.17 0.136	0.09 0.443	-0.00 0.974	0.04 0.743	0.06 0.576	-0.02 0.858	0.10 0.361	0.04 0.737
	lf	-0.01 0.918	-0.10 0.362	-0.10 0.399	0.07 0.550	-0.00 0.967	0.00 0.993	-0.11 0.314	-0.05 0.664
β angle	rf	0.00 0.987	0.12 0.278	0.01 0.917	-0.23 0.042*	-0.23 0.043*	-0.11 0.342	-0.13 0.246	-0.15 0.183
	lf	0.01 0.935	0.07 0.545	-0.03 0.777	-0.11 0.316	-0.06 0.625	-0.19 0.092	-0.05 0.646	-0.11 0.314

*Abbreviations*: *rf *right foot, *lf* left foot, *R* Spearman rank correlation coefficient, *p* probability value

*p<0.05

The data in Table 6 show statistically significant positive relationships between the variables that determine comfort and most of the characteristics that determine functionality, as well as ratings of attractiveness (style). As subjective ratings of comfort increased, so did ratings of functionality and attractiveness. Only the relationships between perceived heel height, shoe weight and shoe length with breathability were not shown.

**Table 6:** Relationships of comfort perceptions with footwear functionality and attractiveness

Variable	Grip	Durability	Safety	Weight	Breathability	Ease of donning and doffing	Individualiztion	Style
	R p							
Shoe length	0.45 <0.001*	0.43 <0.001*	0.38 0.001*	0.26 0.019*	0.22 0.054	0.40 <0.001*	0.30 0.006*	0.30 0.006*
Shoe forefoot width	0.38 <0.001*	0.29 0.008*	0.34 0.002*	0.35 0.001*	0.24 0.032*	0.42 <0.001*	0.40 <0.001*	0.38 <0.001*
Shoe heel width	0.52 <0.001*	0.23 0.037*	0.49 <0.001*	0.39 <0.001*	0.43 <0.001*	0.56 <0.001*	0.43 <0.001*	0.41 <0.001*
Heel height	0.60 <0.001*	0.34 0.002*	0.55 <0.001*	0.20 0.068	0.29 0.009*	0.38 <0.001*	0.29 0.009*	0.37 0.001*
Heel cushioning	0.40 <0.001*	0.37 0.001*	0.31 0.005*	0.52 <0.001*	0.57 <0.001*	0.38 0.001*	0.60 <0.001*	0.39 <0.001*
Forefoot cushioning	0.53 <0.001*	0.38 0.001*	0.36 0.001*	0.48 <0.001*	0.46 <0.001*	0.37 0.001*	0.70 <0.001*	0.44 <0.001*
Arch height	0.64 <0.001*	0.32 0.004*	0.62 <0.001*	0.36 0.001*	0.40 <0.001*	0.41 <0.001*	0.55 <0.001*	0.49 <0.001*
Mediolateral control	0.63 <0.001*	0.52 <0.001*	0.47 <0.001*	0.42 <0.001*	0.54 <0.001*	0.53 <0.001*	0.56 <0.001*	0.42 <0.001*
Overall comfort	0.46 <0.001*	0.46 <0.001*	0.36 <0.001*	0.53 <0.001*	0.58 <0.001*	0.02 <0.001*	0.78 <0.001*	0.58 <0.001*
Material properties	0.43 <0.001*	0.40 <0.001*	0.41 <0.001*	0.65 <0.001*	0.59 <0.001*	0.49 <0.001*	0.66 <0.001*	0.58 <0.001*

*Abbreviations*: *rf *right foot, *lf* left foot, *R* Spearman rank correlation coefficient, *p* probability value

*p<0.05

## Discussion

The aim of the study was to evaluate foot characteristics as well as perceived comfort, functionality and attractiveness of safety boots and their mutual relationships among production workers. We have shown that the subjective perception of safety boot comfort did not differ between the females or males for any of the ten aspects investigated. Similarly, Nesterovica et al [21] found no gender differences in the subjective evaluation of six comfort aspects of military boots in active duty infantry soldiers of the Latvian Land Forces. The authors concluded that correct sizing is a prerequisite for high comfort ratings. In a study by Janson et al [14], 60% of females and 45% of males industrial workers found safety footwear to be less comfortable than normal everyday shoes, and the level of discomfort accepted for safety footwear was higher than for normal shoes. When asked about the most uncomfortable part of the shoe, females pointed to the nose, while males said the sole. Women used safety footwear for a shorter period of time and changed them more often, during breaks and after work, while males usually wore them all day, including on the way to work. Females tended to change their safety footwear less often, which the authors suggest may be due to less wear and tear due to shorter periods of wear, as well as keeping shoes that they found comfortable for as long as possible. 

Our research showed that females rated individualization (how the shoe fits the foot) lower than men in terms of functionality, and the style of the tested shoes lower than males in terms of attractiveness. These data indicate aspects that are important to females and suggest the need to design and produce shoes that adapt to the different conformations of female and male feet. Shen et al [22] also showed differences in the perception of some shoe features by female and male participants. In addition to features such as forefoot cushioning, breathability, comfort, and durability of the upper, the female gender placed a high value on the color of the shoe. As a result of their research on the preferences of females with rheumatic disease, Naidoo et al [23] similarly indicated that the decision to choose footwear was related to a psychosocial factor, and the female sex was largely influenced by the aesthetic appearance of the shoes. It seems that the lower rating of individualization and attractiveness of shoes may be dictated by the fact that female footwear is a "scaled-down" version of footwear produced for male. Females are therefore forced to wear shoes that fit men's feet. In a previous study, Puszczalowska-Lizis et al [24] highlighted gender differences in foot characteristics, suggesting that the protective footwear industry should move away from the production of "unisex" footwear and focus on individualization/personalization, taking into account both the specific fitting requirements due to the structure of the female foot and the aesthetic preferences of females.

In our study, for the majority of foot anatomy data, there were no statistically significant differences between groups. Therefore, we collapsed the male and female foot structure data for the correlation tests. Perceived comfort of shoe length decreased as foot width increased. This may be due to differences between actual and estimated size, suggesting the need to consider different widths for the same length when designing safety footwear. Our results are consistent with those reported by other authors. Janson et al [14] showed that over 22% of females and 19% of males employed in the UK industrial sector choose a larger shoe size or wear extra socks to adjust shoes to the width of their feet. The authors suggest that there is a need to increase shoe production to accommodate half sizes. Similarly, Dobson et al [25] found that Australian miners were wearing work boots that were much longer than the actual length of their feet. The main reason was that the correct length boots were too narrow in relation to the width of the forefoot. Poorly fitting work boots are not only uncomfortable, but can also cause foot pain during work. Therefore, the authors clearly emphasized that one boot design cannot meet all the work-related requirements of underground coal mining.

In the proprietary material, as the medial longitudinal arch of the foot increased, the perception of comfort in terms of heel cushioning decreased. These results suggest that people with high arches need better cushioning and therefore prefer insoles made of soft, flexible materials. Raising the midfoot (between the heads of the metatarsals and the heel bone) above the ground and increasing the angle between the axis of the heel bone and the plane of the ground makes the foot more rigid and less cushioned. In addition, limited support function, midstance abnormalities, and a possible pronation deficit result in a weaker shoe fit, which in turn determines a decrease in subjective comfort ratings. For comparison, in the study by Anderson et al [20], people with low arches preferred a firm insole, which would be consistent with the fact that they require less cushioning than people with high arches. This suggests that people with foot problems may rate footwear less favorably for certain comfort characteristics. Therefore, footwear made according to certain standards is not adapted to the feet of people with deformations to the foot.

Our study suggests relationships between foot characteristics and subjective perceptions of comfort and functionality of safety footwear. An increase in the longitudinal arch of both feet was associated with lower ratings of the weight, breathability and attractiveness of the footwear, while an increase in the transverse arch of the left foot, which has a supporting function, was associated with lower ratings of the weight of the footwear. These data confirmed our belief that the shape of the foot, while having a direct impact on the fit of the shoe, also affects the perception of comfort, mainly because different areas of the foot have different sensory sensitivities. These observations are in line with a previous study by Puszczalowska-Lizis et al. [24] in the youngest old people, which showed that the perception of shoe comfort in terms of heel cushioning, forefoot cushioning and medial arch height of the insole deteriorated with an increase in the transverse arch of the foot. This suggests that an increase in the arch of the foot reduces the contact area between the foot and the shoe, leading to a lower rating of shoe comfort. 

It is worth noting that people with malaligned arches may experience greater fatigue of the foot muscles, so the use of safety boots, which are already relatively heavy (due to steel toecaps, among other things), may cause an additional sensation of heaviness, hence the lower rating of footwear weight. In our study, the weight and breathability ratings of the footwear also decreased as the fifth toe varus deformity of the right foot increased, confirming the observations of Buldt and Menz [26] that differences in foot morphology may cause problems in selecting appropriate footwear due to the difficulty of fitting into irregular shapes resulting from the deformity. It is also worth noting that the perception of footwear comfort may be related to the steel toe cap. This is particularly true for people with toe deformities and high arches, where despite the usual rubber strip around the edge of the toe cap, it may affect the toes and instep of the foot. Janson et al [14] also showed that the main area of discomfort for people working in safety footwear is where the toes touch the toe cap or where the toe cap ends. According to the authors, traditionally toe caps have not been tailored to a specific size, so most manufacturers of safety footwear use them in a size that covers two and sometimes even three full shoe sizes. As a result, some users find the footwear less comfortable.

Our study showed that as the width of the left foot increased, subjects rated their donning and doffing comfort as worse. This suggests the need to design footwear with flexible uppers that can accommodate the width of both feet, or possibly the need for special work socks. In fact, Stewart et al [27] pointed out that a tight fit causes compression and subsequent discomfort as well as tissue damage and pain. Loose-fitting footwear, on the other hand, is not as uncomfortable, but its wearers may experience other problems, such as blisters caused by friction.

Greatly encouraging results, as yielded by the present study, make a contribution to further research on this subject, indubitably requiring investigation of the already established trends even more thoroughly, correlating the data separately among women and men, and assessing foot shape during dynamic motion, as proposed by Mauch et al. [28] as well as Grau and Barisch-Fritz [29].

As the subjective rating of the comfort of the safety boot increased, so did the rating of its functionality and attractiveness. This suggests that footwear that is more comfortable is also more functional and aesthetically pleasing. Goto and Abe [30] believe that the comfort of footwear is primarily concerned with the ease of wearing, which includes aspects such as correct sizing, fit to the foot, softness and flexibility of materials, and stability and cushioning. Functionality, on the other hand, is primarily concerned with the ability of footwear to perform certain tasks, such as protecting feet from injury, providing adequate traction on the ground, allowing proper breathability or being easy to put on and take off. In the present study, it is indicated that taking both comfort and functionality of footwear into account is justified, because they are interconnected and together determine the overall quality of the product. When footwear is functional, i.e. it supports the foot well, is properly fitted and fulfils its tasks (e.g. provides stability, cushioning or protection), then, the comfort of wearing it increases. In turn, if shoes are comfortable, the user is more willing to wear them, which translates into better functioning in everyday situations, e.g. during work. In this research, it is shown that these two elements complement each other, thus, well-designed footwear must combine both comfort and functionality to meet the expectations of users and provide them with full satisfaction. Therefore, it seems valid that both of these aspects should be simultaneously taken into account when designing and assessing footwear. A further step should be to make appropriate changes so that footwear does not interfere with the proper performance of work-related tasks and, most importantly, the health of employees. This can be achieved by educating people, especially females, about the dangers of ill-fitting work shoes. It is also important to develop recommendations for designers and manufacturers of safety footwear to move away from the production of unisex footwear and the use of female lasts in the production of female footwear. 

## Limitations and future research

The way the subjects were included in the study pursued in line with the adopted inclusion criteria which,on the one hand, allowed to ensure homogeneity within a group, fully corresponding to pertinent characteristics of the 50-60 year-old occupationally active industrial worker population, while on the other, this resulted in reducing the number of potential re-cruits, which may well be regarded as a study limitation. Secondly, the use of the Mündermann comfort scale requires experienced raters, while the Anderson scale for assessing functional aspects of footwear has not yet been evaluated in terms of reliability. Therefore, other research tools may be considered in future studies on the subject. Thirdly, the analysis did not take foot clusters/types into account, which may be a likely reason for not seeing any differences in comfort rating. Measurements of joint girth, arch length, and instep height, as well as assessment of the foot shape during dynamic motion should also be included in further studies. Fourth of all, correlation analysis was performed separately for the right and left foot, while in future research, the results could be averaged for both feet. Additionally, it would be possible to normalise foot length/width to body height or mass to provide results as to whether females have a smaller/slender foot relative to their stature. Given the overall gravity of the issue under study, any subsequent reports would tangibly contribute to its development, while at the same time, granting it due research prominence and much deserved public exposure.

## Conclusions

1. Subjective perception of safety boot comfort did not differ between females and males. However, in terms of functionality, women rated individualization (fitting of footwear to the foot) lower and in terms of attractiveness - the style of the tested footwear. Therefore, recommendations should be developed for designers and manufacturers of safety footwear to move away from the production of unisex footwear and use female shoe lasts to produce female footwear.
2. As the width of the foot increases, the perception of comfort in relation to the length of the shoe has decreased, and this may be due to differences between the actual size and the estimated size. Therefore, different widths for the same length size should be considered when designing safety boots. As the longitudinal arch increased, the perception of comfort in terms of heel cushioning decreased, suggesting that footwear made to certain standards is not adapted to the feet of people with foot deformities. People with high arches need better cushioning and therefore prefer insoles made of soft, flexible materials.An increase in the longitudinal arch of both feet was associated with a lower rating of the weight, breathability and attractiveness of the footwear, while an increase in the transverse arch of the left foot, which has a supporting function, was associated with a lower rating of the weight of the footwear. This suggests that the shape of the foot, which has a direct effect on the fit of the shoe, determines the perception of comfort. As the width of the left foot increased, respondents rated the ease of donning and doffing lower, indicating a need to design safety boots with special flexible uppers to accommodate the width of both feet, or possibly the need for special work socks.
3. More comfortable footwear is also more functional and aesthetically pleasing. Therefore, consideration of these aspects in the design and manufacture of footwear can result in the proper functioning of the worker in the workplace.

## Data Availability

The datasets generated during and/or analysed during the current study are available from the corresponding author on reasonable request.
